# Supplementary material for: Loss-of-function, gain-of-function and dominant-negative mutations have profoundly different effects on protein structure
Source: Nat Commun. 2022 Jul 6;13:3895. doi: 10.1038/s41467-022-31686-6 (PMC9259657; doi:10.1038/s41467-022-31686-6)
Supplement: Supplementary file 1 — Supplementary Information [file 41467_2022_31686_MOESM1_ESM.pdf]

Supplemental figures and tables

Lukas Gerasimavicius, Benjamin J Livesey and Joseph A. Marsh*

MRC Human Genetics Unit, Institute of Genetics & Cancer, University of Edinburgh, Edinburgh, UK

**joseph.marsh@ed.ac.uk*

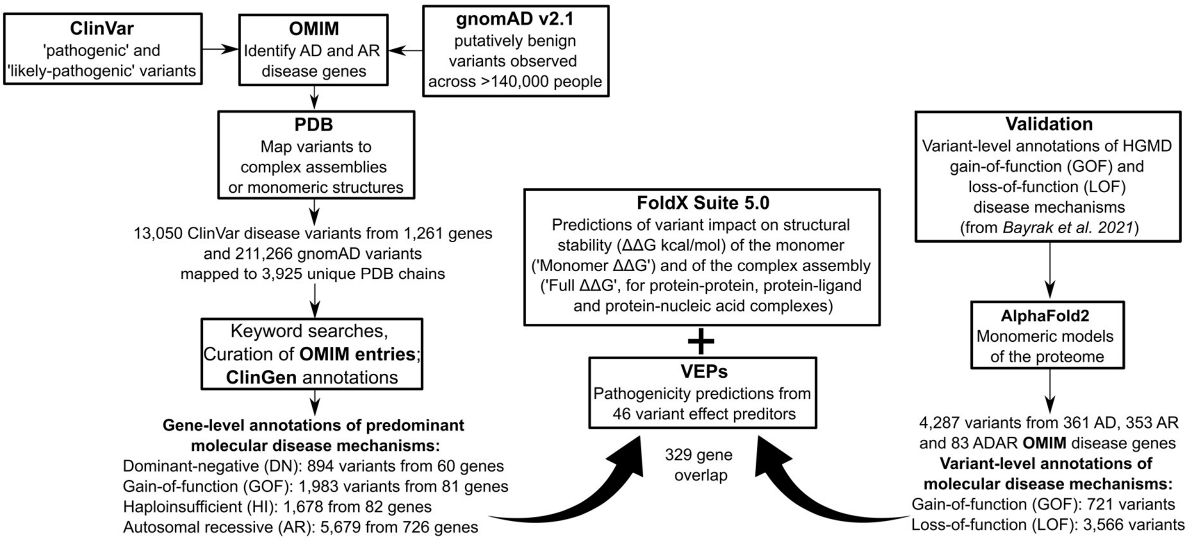

Supplementary Figure 1. **Schematic representation of the data collection, gene annotation, prediction and validation setups.** See 'Methods'.

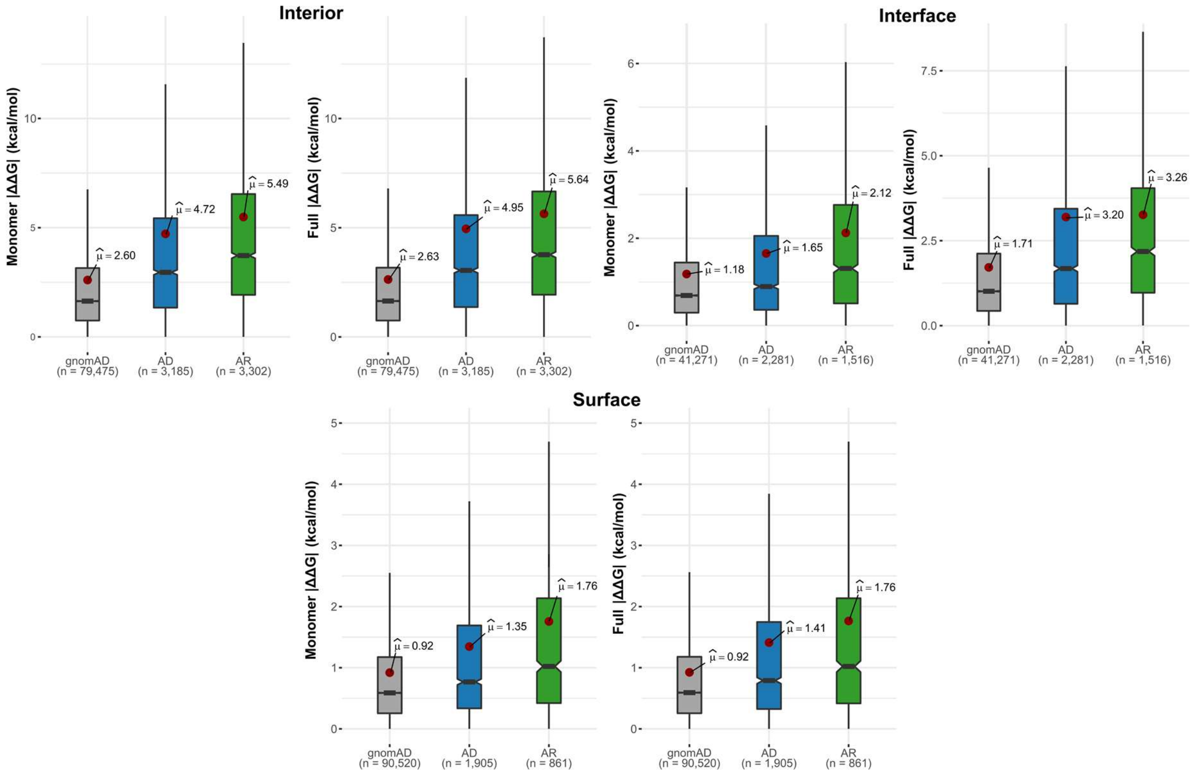

Supplementary Figure 2. **Differences in |ΔΔG| values between gnomAD, autosomal dominant and autosomal recessive mutations are observed across interior, interface and surface locations.** All pairwise group comparisons showed significant differences ($p < 5.0 \times 10^{-6}$, two-sided Holm-corrected Dunn's test).

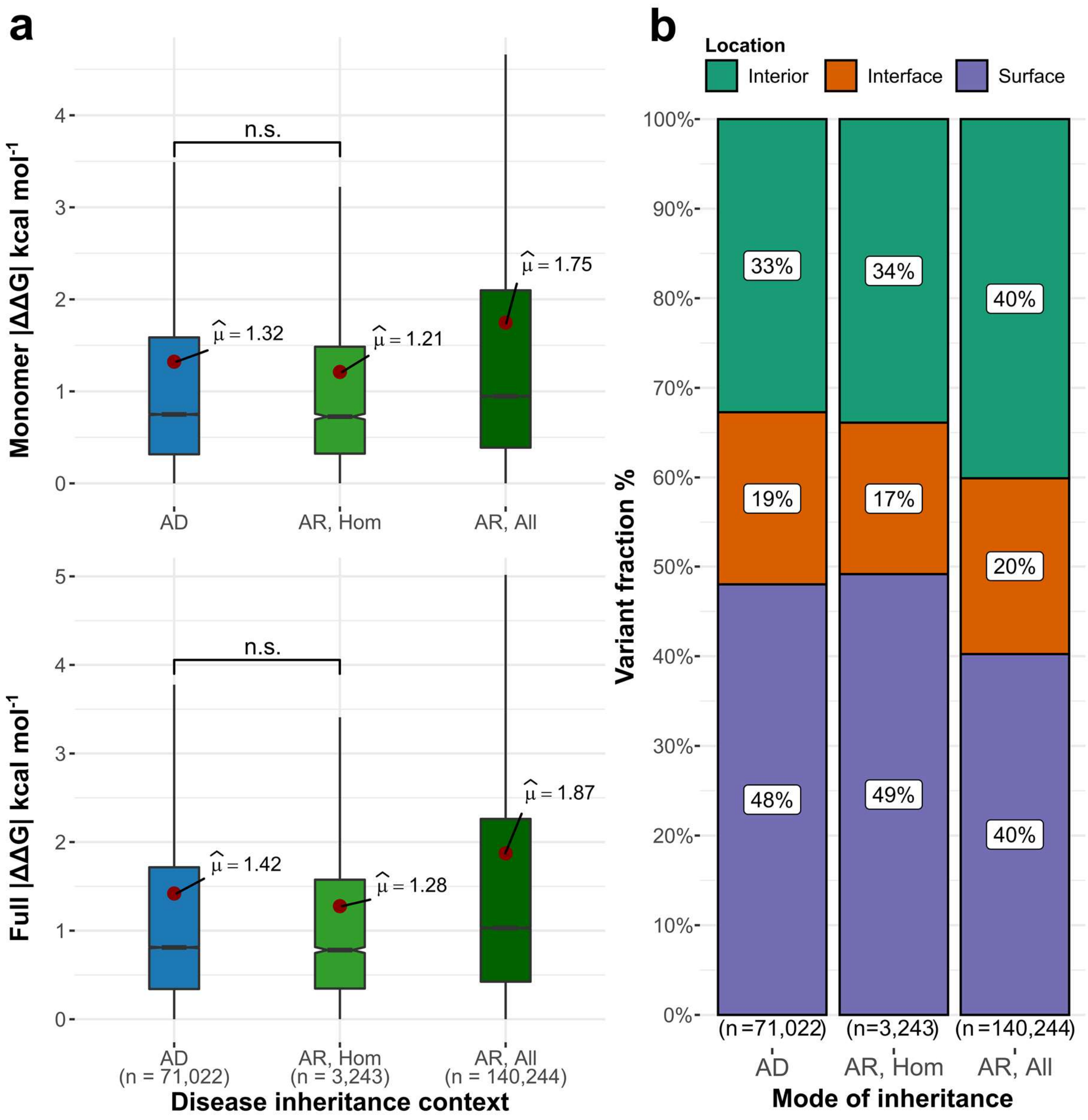

Supplementary Figure 3. **Accounting for variant zygosity reveals highly similar |ΔΔG| values and location distributions for gnomAD variants in autosomal dominant and autosomal recessive disease genes. a** Stability perturbation differences between gnomAD variants of different inheritance contexts and zygosity. 'AR, All' considers all gnomAD variants from the AR genes, while 'AR, Hom' only includes those variants that have been observed in a homozygous state in gnomAD at least once. Boxes denote data within 25th and 75th percentiles, and contain median (middle line) and mean (red dot) value notations. Whiskers extend from the box to furthest values within 1.5x the inter-quartile range. Pairwise group comparisons are significant unless specified ($p < 1.4 \times 10^{-34}$, two-sided Holm-corrected Dunn's test). **b** Proportions of gnomAD variants throughout spatial structure locations for gene variants characterised by different inheritance context groups (all Chi-square comparisons are significant; Cramer's *V* effect sizes are 0.01 and 0.08 for AD vs AR comparisons, using only homozygous recessive variants or all recessive variants, respectively).

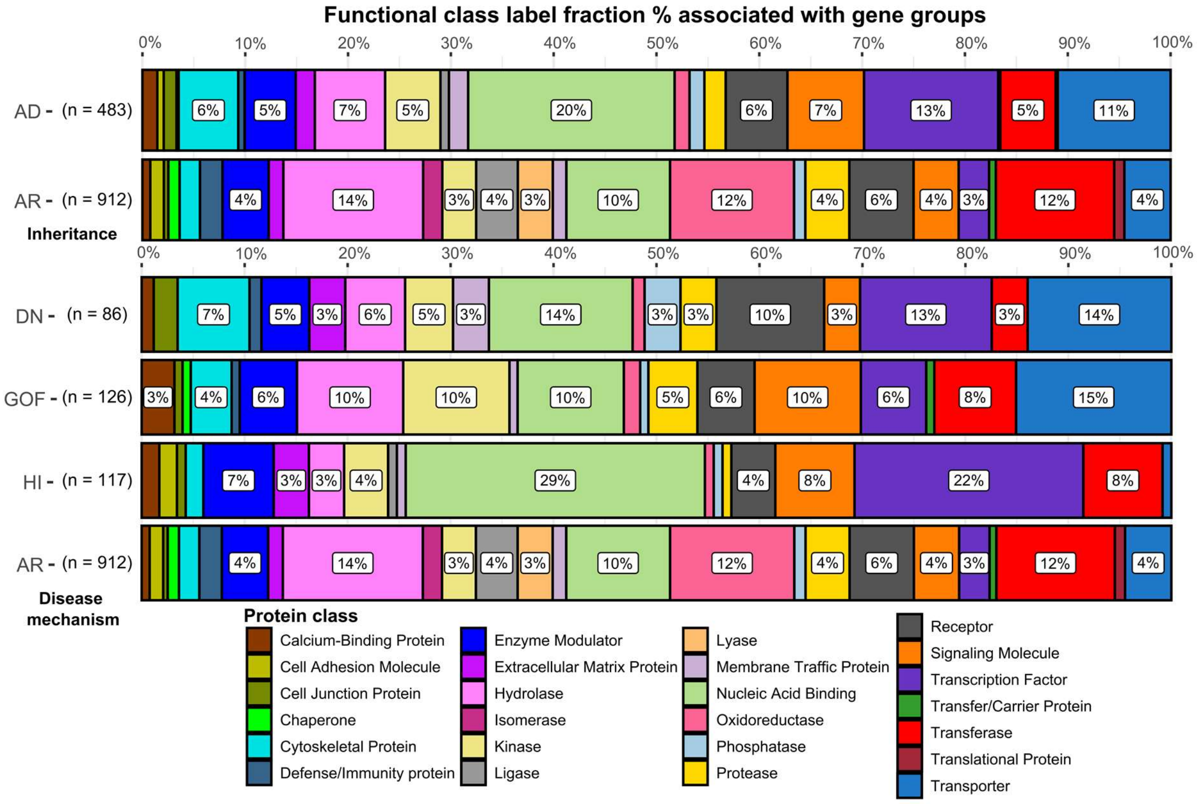

Supplementary Figure 4. **Inheritance and molecular mechanism gene groups are characterised by distinct functional protein class label prevalence.** Chi-square test comparisons were performed within the inheritance and mechanism groups, with only the non-LOF DN *vs* GOF mechanism genes showing insufficient difference ($p = 0.147$, highest significant $p = 4.116 \times 10^{-3}$ after Holm's correction for multiple comparisons). Sample sizes denote functional class label number. The same gene can be associated with multiple functional classes.

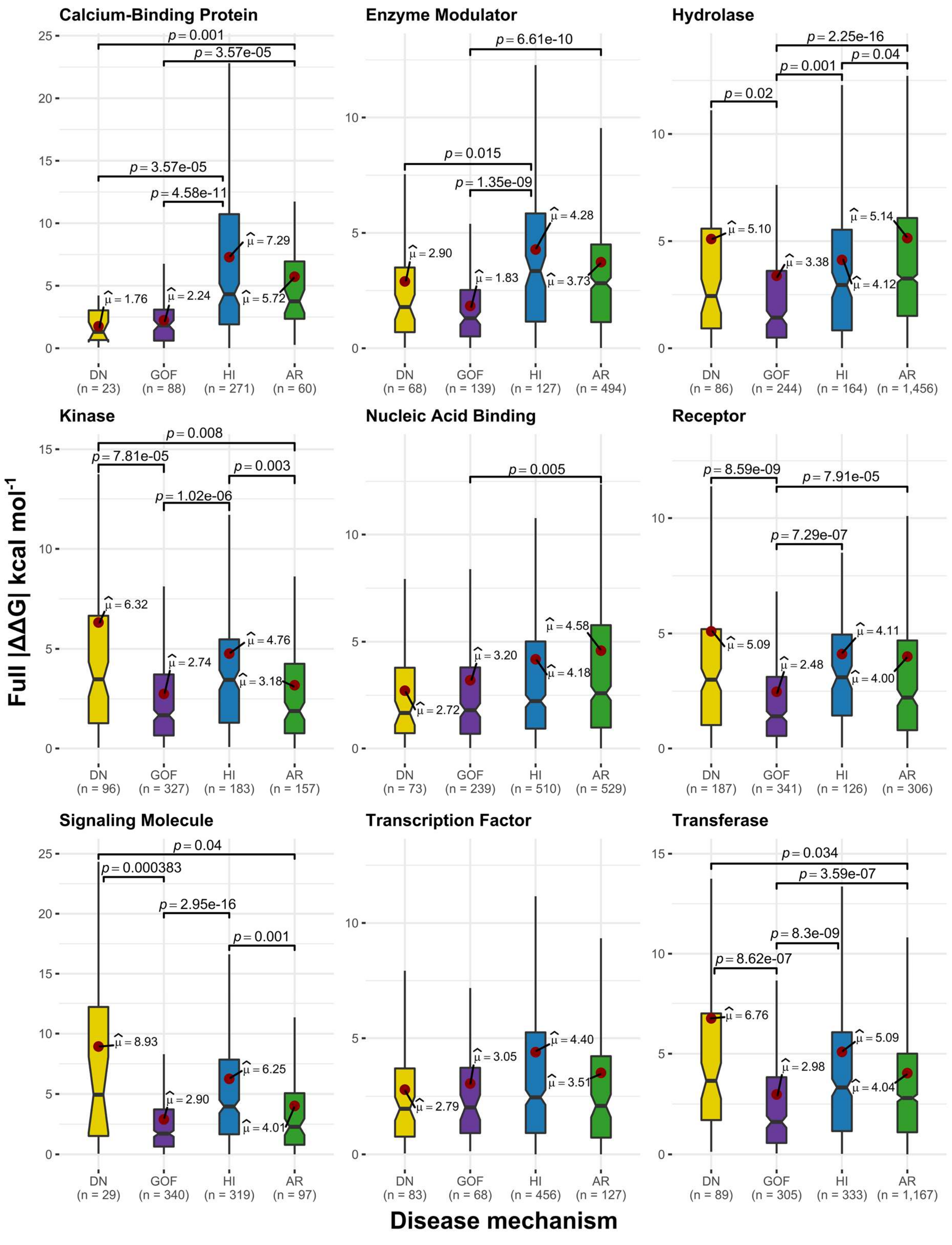

Supplementary Figure 5. **Underlying functional protein class does not necessarily drive the observed variance differences in distinct molecular mechanism perturbation magnitude**. Boxes denote data within 25th and 75th percentiles, and contain median (middle line) and mean (red dot) value notations. Whiskers extend from the box to furthest values within 1.5x the inter-quartile range. Only functional class groups with at least 20 variants in each molecular mechanism were analysed. Statistically significant comparisons are shown (two-sided Holm-corrected Dunn's test).

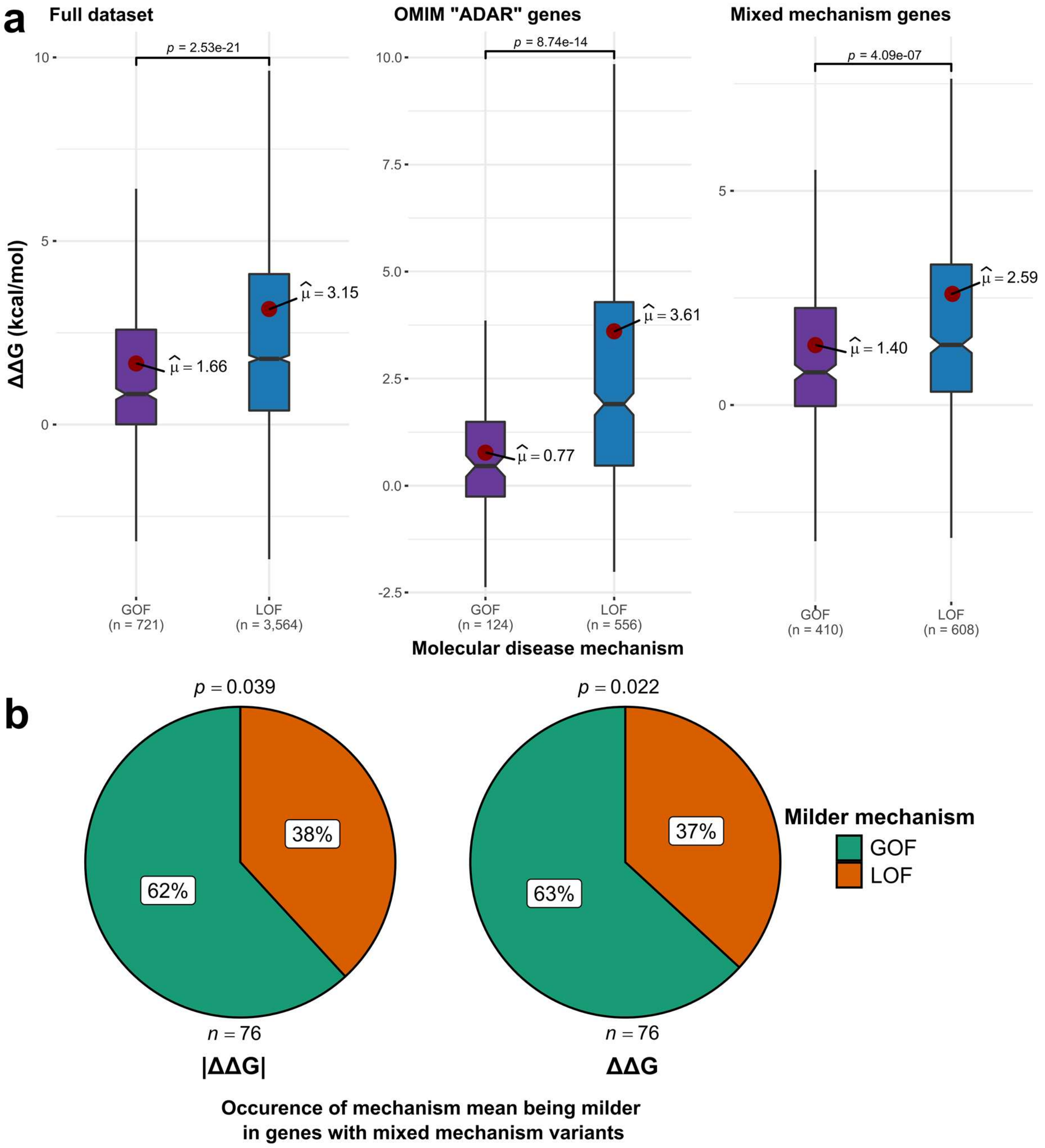

Supplementary Figure 6. **Gain-of-function variants are also milder than loss-of-function mutations in mixed inheritance and mechanism genes.** The variant-level validation dataset is based on the HGMD GOF/LOF disease mechanism data from Bayrak et al., 2021[1]. **a** Comparison of predicted absolute ΔΔG value differences between GOF and LOF variants in several distinct contexts: the full dataset; only looking at mixed inheritance OMIM 'ADAR' genes; only genes with both GOF and LOF variants. Boxes denote data within 25th and 75th percentiles, and contain median (middle line) and mean (red dot) value notations. Whiskers extend from the box to furthest values within 1.5x the inter-quartile range. Significant group comparisons are denoted (two-sided Wilcoxon rank-sum test). **b** Proportion of genes with both kind of variants (GOF & LOF), according to which mechanism group demonstrates a lower predicted ΔΔG mean within the same gene (Chi-square *p*-values depicted).

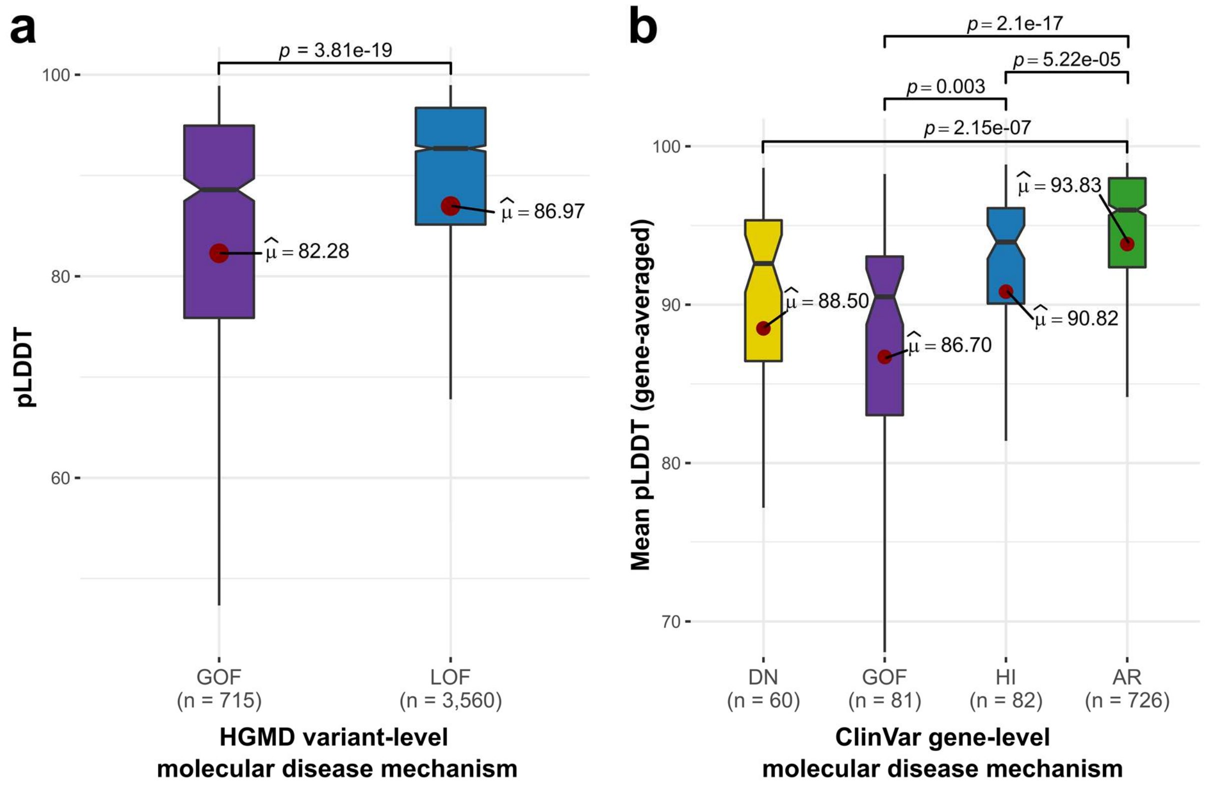

Supplementary Figure 7. **Gain-of-function variants occur in less structurally ordered positions than other mechanism mutations, according to the AlphaFold pLDDT modelling quality metric.** The AlphaFold pLDDT metric has been shown to be an accurate proxy for structural disorder[2]. Boxes denote data within 25th and 75th percentiles, and contain median (middle line) and mean (red dot) value notations. Whiskers extend from the box to furthest values within 1.5x the inter-quartile range. Statistically significant comparisons are shown (two-sided Holm-corrected Dunn's test). **a** pLDDT differences between HGMD GOF and LOF variants. Sample sizes represent variant number. **b** pLDDT comparison across gene-level ClinVar mechanism groups. To control for gene-level annotation biases and uneven variant counts across genes, the pLDDT value is presented as a per-gene mean. Sample sizes represent gene number.

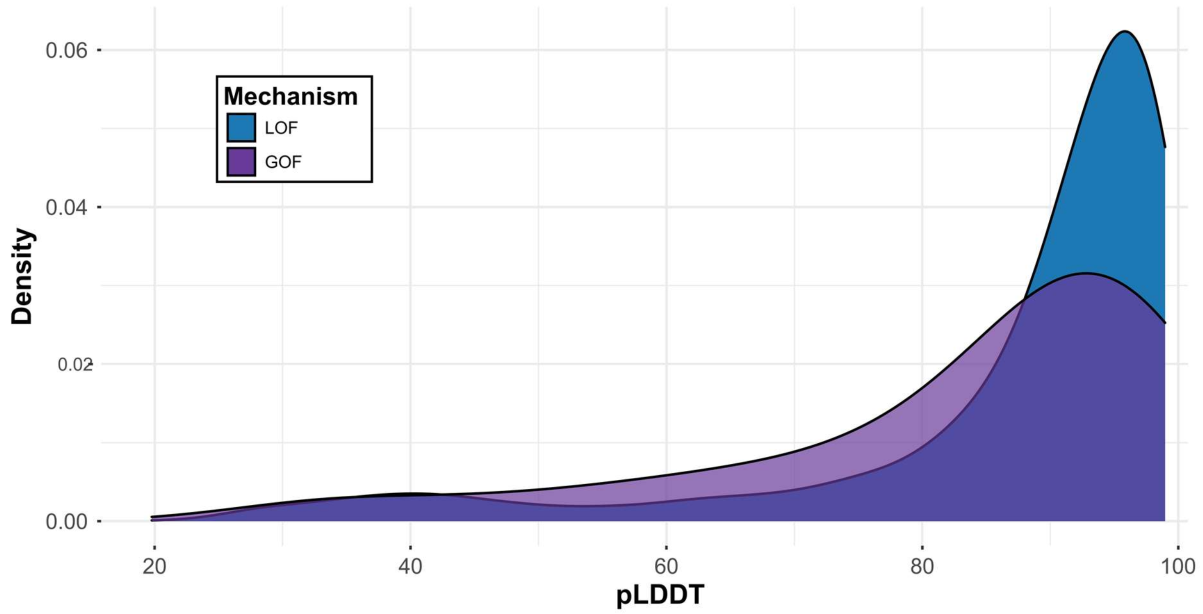

Supplementary Figure 8. **Majority of disease variants occur in positions characterised by high pLDDT, independent of variant-level mechanism.** The values were derived using the variant-level HGMD dataset.

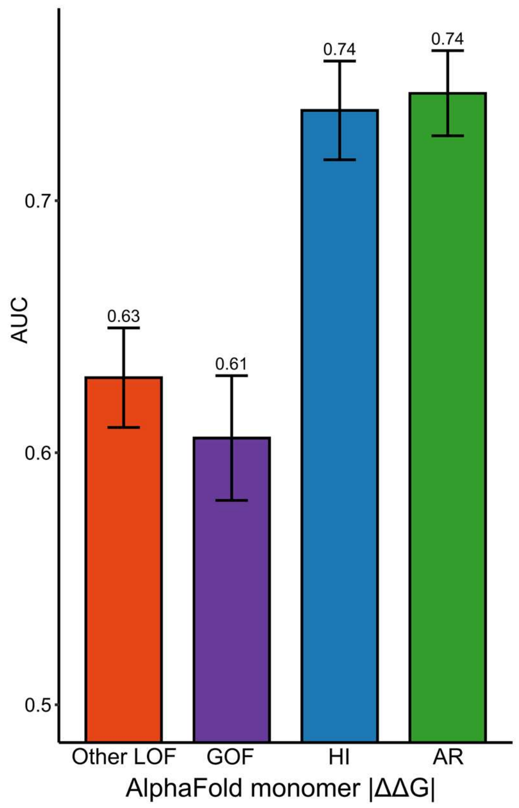

Supplementary Figure 9. **Independent dataset recapitulates observed non-LOF mechanism structural mildness.** The variant-level validation dataset is based on the HGMD GOF/LOF disease mechanism data from Bayrak et al., 2021[1]. AUC values calculated from ROC curves for discriminating between different types of pathogenic HGMD mutations, and putatively benign gnomAD variants, based on predicted stability change score. Only homozygous gnomAD variants were included for the AR analysis. Error bars denote 95% confidence intervals.

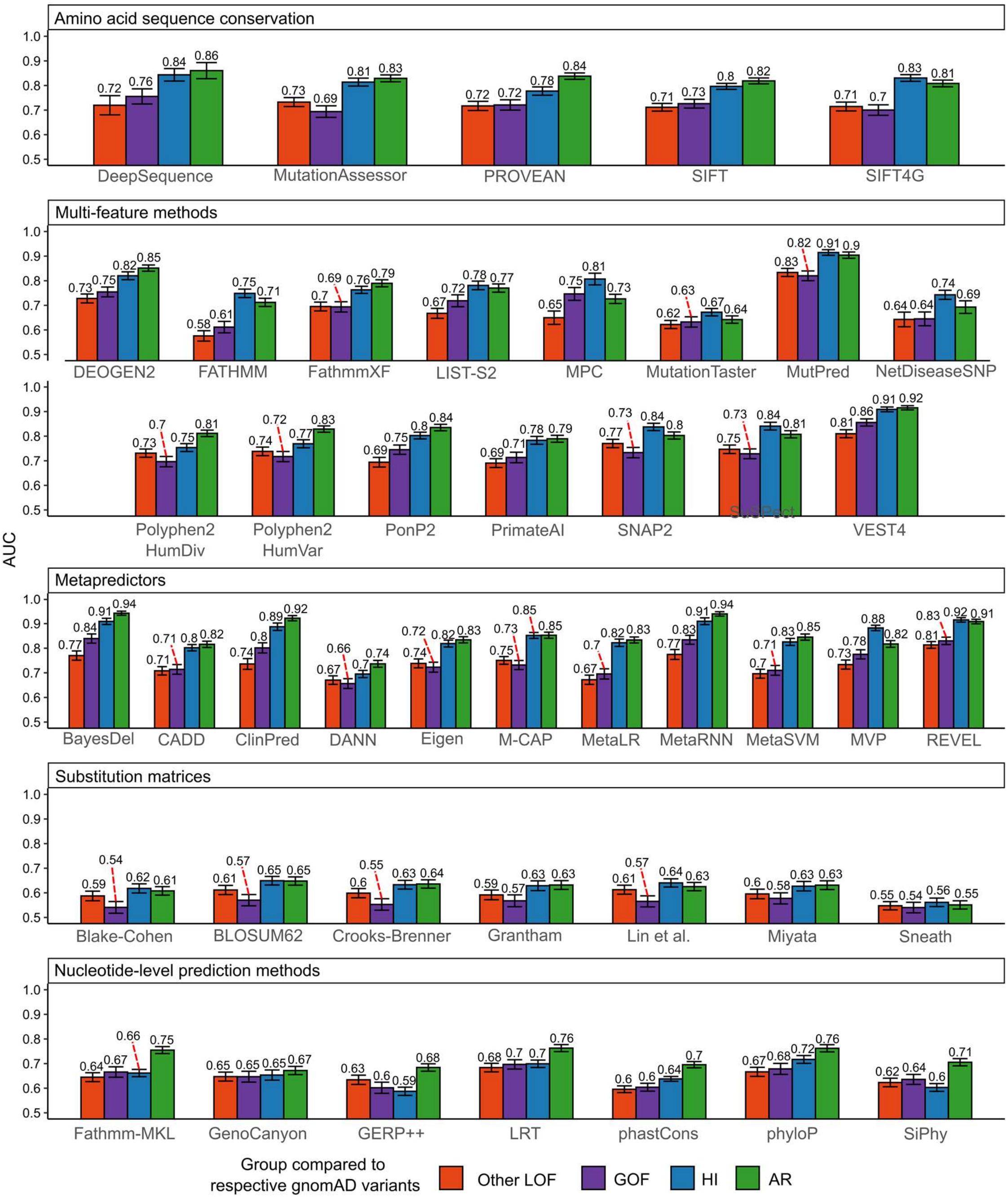

Supplementary Figure 10. **Hybrid variant- and gene-level disease mechanism classification approach validates previously observed VEP performance results.** The validation dataset is based on an external variant-level disease mechanism GOF/LOF label dataset from Bayrak et al. 2021[1] (see 'Methods'). AUC values calculated from ROC curves for discriminating between different types of pathogenic HGMD mutations and putatively benign gnomAD variants, using the outputs of different computational variant effect predictors. Only homozygous gnomAD variants were included for the AR analysis. Error bars denote 95% confidence intervals.

Supplementary Table 1. **Optimal FoldX |ΔΔG| thresholds to distinguish between pathogenic and putatively benign mutations for genes associated with different molecular disease mechanisms.**

Molecular disease mechanism group	Predictor	Optimal threshold (kcal/mol)	Specificity (low est.)	Specificity (median)	Specificity (high est.)	Sensitivity (low est.)	Sensitivity (median)	Sensitivity (high est.)
DN	FoldX Monomer |ΔΔG|	1.03	52.01	55.15	58.39	60.46	61.78	63.06
GOF	FoldX Monomer |ΔΔG|	0.98	56.68	58.80	60.97	57.88	58.72	59.58
HI	FoldX Monomer |ΔΔG|	1.38	62.22	64.66	66.92	72.76	73.63	74.54
AR (homozygous gnomAD)	FoldX Monomer |ΔΔG|	1.41	64.61	65.87	67.14	72.12	73.64	75.21
DN	FoldX Full |ΔΔG|	1.28	57.05	60.18	63.53	64.70	65.95	67.29
GOF	FoldX Full |ΔΔG|	1.16	55.87	57.89	60.01	61.29	62.06	62.84
HI	FoldX Full |ΔΔG|	1.59	66.03	68.30	70.50	74.81	75.65	76.52
AR (homozygous gnomAD)	FoldX Full |ΔΔG|	1.55	67.11	68.30	69.50	73.27	74.68	76.19

Supplementary Table 2. **Variant effect predictors used in this study.**

Predictor	Feature-based classification	ClinVar disease dataset size	gnomAD dataset size	Source access	Reference
BayesDel	Metapredictor	5017	39289	dbNSFP database	(Feng, B. J., 2017)[3]
Blake-Cohen	Substitution matrix	13048	211134	https://www.genome.jp/entry/aaindex:BLAJ010101	(Blake & Cohen, 2001)[4]
BLOSUM62	Substitution matrix	13048	211134	https://www.genome.jp/entry/aaindex:HENS920102	(Henikoff & Henikoff, 1992)[5]
CADD	Metapredictor	12806	208522	https://cadd.gs.washington.edu/snv	(Kircher et al., 2014)[6]
ClinPred	Metapredictor	5017	39289	dbNSFP database	(Alirezaie et al., 2018)[7]
Crooks-Brenner	Substitution matrix	13048	211134	https://www.genome.jp/entry/aaindex:CROG050101	(Crooks & Brenner, 2005)[8]
DANN	Metapredictor	12801	208138	dbNSFP database	(Quang et al., 2015)[9]
DeepSequence	Amino acid sequence conservation	4613	19029	https://github.com/debbiemarkslab/DeepSequence	(Riesselman et al., 2018)[10]
DEOGEN2	Multi-feature	12095	202031	https://deogen2.mutaframe.com/	(Raimondi et al., 2017)[11]
Eigen	Metapredictor	12772	207420	dbNSFP database	(Ionita-Laza et al., 2016)[12]
FATHMM	Multi-feature	12113	196893	http://fathmm.biocompute.org.uk/inherited.html	(Shihab et al., 2013)[13]
Fathmm-MKL	Multi-feature	12801	208138	dbNSFP database	(Shihab et al., 2015)[14]
FathmmXF	Nucleotide-level prediction method	12712	207661	http://fathmm.biocompute.org.uk/fathmm-xf/	(Rogers et al., 2018)[15]
GenoCanyon	Nucleotide-level prediction method	12801	208138	dbNSFP database	(Lu et al., 2015)[16]
GERP++	Nucleotide-level prediction method	12801	208129	dbNSFP database	(Davydov et al., 2010)[17]
Grantham	Substitution matrix	13048	211134	https://www.genome.jp/entry/aaindex:GRAR740104	(Grantham, 1974)[18]
Lin et al.	Substitution matrix	13048	211134	https://www.genome.jp/entry/aaindex:LINK010101	(Lin et al., 2001)[19]
LIST-S2	Metapredictor	4407	34468	dbNSFP database	(Malhis et al., 2020)[20]
LRT	Nucleotide-level prediction method	12546	201271	dbNSFP database	(Chun & Fay, 2009)[21]
M-CAP	Metapredictor	12746	206078	http://bejerano.stanford.edu/mcap/	(Jagadeesh et al., 2016)[22]
MetaLR	Metapredictor	12772	207420	dbNSFP database	(Dong et al., 2015)[23]
MetaRNN	Metapredictor	5017	39289	dbNSFP database	(Li et al., 2021)[24]
MetaSVM	Metapredictor	12772	207420	dbNSFP database	(Dong et al., 2015)[23]

Miyata	Substitution matrix	13048	211134	https://www.genome.jp/entry/aaindex:MIYT790101	(Miyata et al., 1979)[25]
MPC	Multi-feature	9088	162846	dbNSFP database	(Samocha et al., 2017)[26]
MutationAssessor	Amino acid sequence conservation	11873	195899	dbNSFP database	(Reva et al., 2011)[27]
MutationTaster	Multi-feature	12761	206319	dbNSFP database	(Schwarz et al., 2014)[28]
MutPred	Multi-feature	11554	170575	dbNSFP database	(Pejaver et al., 2020)[29]
MVP	Metapredictor	12528	205851	dbNSFP database	(Qi et al., 2018)[30]
NetDiseaseSNP	Multi-feature	12789	205379	http://www.cbs.dtu.dk/services/NetDiseaseSNP/	(Johansen et al., 2013)[31]
phastCons	Nucleotide-level prediction method	12806	208522	dbNSFP database	(Siepel et al., 2005)[32]
phyloP	Nucleotide-level prediction method	12806	208522	http://papi.unipv.it/	(Pollard et al., 2010)[33]
PolyPhen2 HumDiv	Multi-feature	11771	198850	http://genetics.bwh.harvard.edu/pph2/	(Adzhubei et al., 2010)[34]
PolyPhen2 HumVar	Multi-feature	11771	198850	http://genetics.bwh.harvard.edu/pph2/	(Adzhubei et al., 2010)[34]
PonP2	Multi-feature	12134	196040	http://structure.bmc.lu.se/PON-P2/	(Niroula et al., 2015)[35]
PrimateAI	Multi-feature	12638	206477	dbNSFP database	(Sundaram et al., 2018)[36]
PROVEAN	Amino acid sequence conservation	12044	197456	http://provean.jcvi.org/index.php	(Choi et al., 2012)[37]
REVEL	Metapredictor	12772	207420	https://sites.google.com/site/revelgenomics/	(Ioannidis et al., 2016)[38]
SIFT	Amino acid sequence conservation	12851	208985	https://sift.bii.a-star.edu.sg/www/code.html	(Sim et al., 2012)[39]
SIFT4G	Amino acid sequence conservation	12418	203379	dbNSFP database	(Vaser et al., 2016)[40]
SiPhy	Nucleotide-level prediction method	12799	208117	dbNSFP	(Garber et al., 2009)[41]
SNAP2	Multi-feature	13009	210582	https://www.rostlab.org/services/snap/	(Hecht et al., 2015)[42]
Sneath	Substitution matrix	13048	211134	(Sneath, 1966)	(Sneath, 1966)[43]
SuSPect	Multi-feature	13007	210532	http://www.sbg.bio.ic.ac.uk/suspect/about.html	(Yates et al., 2014)[44]
VEST4	Multi-feature	12633	206645	https://www.cravat.us/CRAVAT/	(Carter et al., 2013)[45]

References

1. Sevim Bayrak, C. *et al.* Identification of discriminative gene-level and protein-level features associated with pathogenic gain-of-function and loss-of-function variants. *Am. J. Hum. Genet.* **108**, 2301–2318 (2021).
2. Tunyasuvunakool, K. *et al.* Highly accurate protein structure prediction for the human proteome. *Nature* **596**, 590–596 (2021).
3. Feng, B.-J. PERCH: A Unified Framework for Disease Gene Prioritization: HUMAN MUTATION. *Hum. Mutat.* **38**, 243–251 (2017).
4. Blake, J. D. & Cohen, F. E. Pairwise sequence alignment below the twilight zone. *J. Mol. Biol.* **307**, 721–735 (2001).
5. Henikoff, S. & Henikoff, J. G. Amino acid substitution matrices from protein blocks. *Proc. Natl. Acad. Sci. U. S. A.* **89**, (1992).
6. Kircher, M. *et al.* A general framework for estimating the relative pathogenicity of human genetic variants. *Nat. Genet.* **46**, 310–315 (2014).
7. Alirezaie, N., Kernohan, K. D., Hartley, T., Majewski, J. & Hocking, T. D. ClinPred: Prediction Tool to Identify Disease-Relevant Nonsynonymous Single-Nucleotide Variants. *Am. J. Hum. Genet.* **103**, 474–483 (2018).
8. Crooks, G. E. & Brenner, S. E. An alternative model of amino acid replacement. *Bioinformatics* **21**, 975–980 (2005).
9. Quang, D., Chen, Y. & Xie, X. DANN: A deep learning approach for annotating the pathogenicity of genetic variants. *Bioinformatics* **31**, 761–763 (2015).
10. Riesselman, A. J., Ingraham, J. B. & Marks, D. S. Deep generative models of genetic variation capture the effects of mutations. *Nat. Methods* **15**, 816–822 (2018).
11. Raimondi, D. *et al.* DEOGEN2: Prediction and interactive visualization of single amino acid variant deleteriousness in human proteins. *Nucleic Acids Res.* **45**, W201–W206 (2017).
12. Ionita-Laza, I., Mccallum, K., Xu, B. & Buxbaum, J. D. A spectral approach integrating functional genomic annotations for coding and noncoding variants. *Nat. Genet.* **48**, 214–220 (2016).
13. Shihab, H. A. *et al.* Predicting the Functional, Molecular, and Phenotypic Consequences of Amino Acid Substitutions using Hidden Markov Models. *Hum. Mutat.* **34**, 57–65 (2013).
14. Shihab, H. A. *et al.* An integrative approach to predicting the functional effects of non-coding and coding sequence variation. *Bioinformatics* **31**, 1536–1543 (2015).
15. Rogers, M. F. *et al.* FATHMM-XF: Accurate prediction of pathogenic point mutations via extended features. *Bioinformatics* **34**, 511–513 (2018).
16. Lu, Q. *et al.* A statistical framework to predict functional non-coding regions in the human genome through integrated analysis of annotation data. *Sci. Rep.* **5**, 1–13 (2015).
17. Davydov, E. V. *et al.* Identifying a high fraction of the human genome to be under selective constraint using GERP++. *PLoS Comput. Biol.* **6**, (2010).
18. Grantham, R. Amino acid difference formula to help explain protein evolution. *Science* **185**, 862–864 (1974).
19. Lin, K., May, A. C. W. & Taylor, W. R. Amino acid substitution matrices from an artificial neural network model. *J. Comput. Biol.* **8**, 471–481 (2001).
20. Malhis, N., Jacobson, M., Jones, S. J. M. & Gsponer, J. LIST-S2: taxonomy based sorting of deleterious missense mutations across species. *Nucleic Acids Res.* **48**, W154–W161 (2020).
21. Chun, S. & Fay, J. C. Identification of deleterious mutations within three human genomes. *Genome Res.* **19**, 1553–1561 (2009).
22. Jagadeesh, K. A. *et al.* M-CAP eliminates a majority of variants of uncertain significance in clinical exomes at high sensitivity. *Nat. Genet.* **48**, 1581–1586 (2016).
23. Dong, C. *et al.* Comparison and integration of deleteriousness prediction methods for nonsynonymous SNVs in whole exome sequencing studies. *Hum. Mol. Genet.* **24**, 2125–2137 (2015).

24. Li, C., Zhi, D., Wang, K. & Liu, X. *MetaRNN: Differentiating Rare Pathogenic and Rare Benign Missense SNVs and InDels Using Deep Learning*. http://biorxiv.org/lookup/doi/10.1101/2021.04.09.438706 (2021) doi:10.1101/2021.04.09.438706.
25. Miyata, T., Miyazawa, S. & Yasunaga, T. Two types of amino acid substitutions in protein evolution. *J. Mol. Evol.* **12**, 219–236 (1979).
26. Samocha, K. E. *et al.* Regional missense constraint improves variant deleteriousness prediction. *bioRxiv* (2017) doi:10.1101/148353.
27. Reva, B., Antipin, Y. & Sander, C. Predicting the functional impact of protein mutations: Application to cancer genomics. *Nucleic Acids Res.* **39**, 37–43 (2011).
28. Schwarz, J. M., Cooper, D. N., Schuelke, M. & Seelow, D. Mutationtaster2: Mutation prediction for the deep-sequencing age. *Nat. Methods* **11**, 361–362 (2014).
29. Pejaver, V. *et al.* Inferring the molecular and phenotypic impact of amino acid variants with MutPred2. *Nat. Commun.* **11**, (2020).
30. Qi, H. *et al.* MVP predicts the pathogenicity of missense variants by deep learning. *Nat. Commun.* **12**, (2021).
31. Johansen, M. B., Izarzugaza, J. M. G., Brunak, S., Petersen, T. N. & Gupta, R. Prediction of Disease Causing Non-Synonymous SNPs by the Artificial Neural Network Predictor NetDiseaseSNP. *PLoS ONE* **8**, (2013).
32. Siepel, A. *et al.* Evolutionarily conserved elements in vertebrate, insect, worm, and yeast genomes. *Genome Res.* **15**, 1034–1050 (2005).
33. Pollard, K. S., Hubisz, M. J., Rosenbloom, K. R. & Siepel, A. Detection of nonneutral substitution rates on mammalian phylogenies. *Genome Res.* **20**, 110–121 (2010).
34. Adzhubei, I. A. *et al.* A method and server for predicting damaging missense mutations. *Nat. Methods* **7**, 248–249 (2010).
35. Niroula, A., Urolagin, S. & Vihinen, M. PON-P2: Prediction method for fast and reliable identification of harmful variants. *PLoS ONE* **10**, 1–17 (2015).
36. Sundaram, L. *et al.* Predicting the clinical impact of human mutation with deep neural networks. *Nat. Genet.* **50**, 1161–1170 (2018).
37. Choi, Y., Sims, G. E., Murphy, S., Miller, J. R. & Chan, A. P. Predicting the Functional Effect of Amino Acid Substitutions and Indels. *PLoS ONE* **7**, (2012).
38. Ioannidis, N. M. *et al.* REVEL: An Ensemble Method for Predicting the Pathogenicity of Rare Missense Variants. *Am. J. Hum. Genet.* **99**, 877–885 (2016).
39. Sim, N. L. *et al.* SIFT web server: Predicting effects of amino acid substitutions on proteins. *Nucleic Acids Res.* **40**, 452–457 (2012).
40. Vaser, R., Adusumalli, S., Leng, S. N., Sikic, M. & Ng, P. C. SIFT missense predictions for genomes. *Nat. Protoc.* **11**, 1–9 (2016).
41. Garber, M. *et al.* Identifying novel constrained elements by exploiting biased substitution patterns. *Bioinformatics* **25**, 54–62 (2009).
42. Hecht, M., Bromberg, Y. & Rost, B. Better prediction of functional effects for sequence variants. *BMC Genomics* **16**, (2015).
43. Sneath, P. H. A. Relations between chemical structure and biological activity in peptides. *J. Theor. Biol.* **12**, 157–195 (1966).
44. Yates, C. M., Filippis, I., Kelley, L. A. & Sternberg, M. J. E. SuSPect: Enhanced prediction of single amino acid variant (SAV) phenotype using network features. *J. Mol. Biol.* **426**, 2692–2701 (2014).
45. Carter, H., Douville, C., Stenson, P. D., Cooper, D. N. & Karchin, R. Identifying Mendelian disease genes with the variant effect scoring tool. *BMC Genomics* **14 Suppl 3**, (2013).
