# Supplementary material for: Loss-of-function, gain-of-function and dominant-negative mutations have profoundly different effects on protein structure
Source: Nat Commun. 2022 Jul 6;13:3895. doi: 10.1038/s41467-022-31686-6 (PMC9259657; doi:10.1038/s41467-022-31686-6)
Supplement: Supplementary file 2 — Reporting Summary [file 41467_2022_31686_MOESM2_ESM.pdf]

nature portfolio

Corresponding author(s): Joseph A. Marsh

Last updated by author(s): Jun 27, 2022

Reporting Summary

Nature Portfolio wishes to improve the reproducibility of the work that we publish. This form provides structure for consistency and transparency in reporting. For further information on Nature Portfolio policies, see our Editorial Policies and the Editorial Policy Checklist.

Statistics

For all statistical analyses, confirm that the following items are present in the figure legend, table legend, main text, or Methods section.

n/a	Confirmed	
☐	☒	The exact sample size (*n*) for each experimental group/condition, given as a discrete number and unit of measurement
☐	☒	A statement on whether measurements were taken from distinct samples or whether the same sample was measured repeatedly
☐	☒	The statistical test(s) used AND whether they are one- or two-sided<br>*Only common tests should be described solely by name; describe more complex techniques in the Methods section.*
☐	☒	A description of all covariates tested
☐	☒	A description of any assumptions or corrections, such as tests of normality and adjustment for multiple comparisons
☐	☒	A full description of the statistical parameters including central tendency (e.g. means) or other basic estimates (e.g. regression coefficient) AND variation (e.g. standard deviation) or associated estimates of uncertainty (e.g. confidence intervals)
☐	☒	For null hypothesis testing, the test statistic (e.g. *F*, *t*, *r*) with confidence intervals, effect sizes, degrees of freedom and *P* value noted<br>*Give P values as exact values whenever suitable.*
☒	☐	For Bayesian analysis, information on the choice of priors and Markov chain Monte Carlo settings
☒	☐	For hierarchical and complex designs, identification of the appropriate level for tests and full reporting of outcomes
☐	☒	Estimates of effect sizes (e.g. Cohen's *d*, Pearson's *r*), indicating how they were calculated

Our web collection on statistics for biologists contains articles on many of the points above.

Software and code

Policy information about availability of computer code

Data collection	Code to calculate our mutation clustering metric from PDB files is available at https://doi.org/10.5281/zenodo.6759338. Stability predictions were derived using FoldX 5.0 (https://foldxsuite.crg.eu/). Gene functional class annotation was updated using PANTHER 7.0 (http://www.pantherdb.org/). Tools used for variant effect prediction: dbNSFP v4.0 (http://database.liulab.science/dbNSFP); PonP2 (http://structure.bmc.lu.se/PON-P2/); SNAP2 (https://www.rostlab.org/services/snap/); SuSPect (http://www.sbg.bio.ic.ac.uk/suspect/); NetDiseaseSNP v1.0 (https://services.healthtech.dtu.dk/service.php?NetDiseaseSNP-1.0); SIFT (https://sift.bii.a-star.edu.sg/), DeepSequence (https://github.com/debbiemarkslab/DeepSequence).
Data analysis	Data analysis was performed in R (version 4.0.4). Additional R packages used for statistical testing included 'pROC' (version 1.17.0.1), 'rmngb' (version 0.6-1), 'binom' (version 1.1-1), 'ggstatsplot' (version 0.7.2), 'rcompanion' (version 2.4.0).

For manuscripts utilizing custom algorithms or software that are central to the research but not yet described in published literature, software must be made available to editors and reviewers. We strongly encourage code deposition in a community repository (e.g. GitHub). See the Nature Portfolio guidelines for submitting code & software for further information.

Data

Policy information about availability of data

All manuscripts must include a data availability statement. This statement should provide the following information, where applicable:

- Accession codes, unique identifiers, or web links for publicly available datasets
- A description of any restrictions on data availability
- For clinical datasets or third party data, please ensure that the statement adheres to our policy

The dataset generated and analysed during the current study is available at https://doi.org/10.17605/OSF.IO/H62FQ. Databases/datasets used in this study:

March 2021

gnomAD (https://gnomad.broadinstitute.org/downloads); ClinVar (https://ftp.ncbi.nlm.nih.gov/pub/clinvar/); dbNSFP v4.0 (http://database.liulab.science/dbNSFP); Itan Lab's GOF/LOF database (https://itanlab.shinyapps.io/goflof/); OMIM (https://www.omim.org/); ClinGen Dosage Sensitivity database (https://search.clinicalgenome.org/kb/gene-dosage), Protein Data Bank (https://www.rcsb.org/).

Field-specific reporting

Please select the one below that is the best fit for your research. If you are not sure, read the appropriate sections before making your selection.

- [x] Life sciences
- [ ] Behavioural & social sciences
- [ ] Ecological, evolutionary & environmental sciences

For a reference copy of the document with all sections, see nature.com/documents/nr-reporting-summary-flat.pdf

Life sciences study design

All studies must disclose on these points even when the disclosure is negative.

Sample size	Sample sizes were not pre-calculated owing to the big-data nature of human genetic variation. All available data, meeting our criteria, was using in the generation and analysis of the dataset.
Data exclusions	All data filtering and exclusion criteria are laid out in the methods section and were pre-established.<br><br>Any variants present in the ClinVar and HGMD datasets were excluded from the gnomAD variant set for respective analyses. For certain analyses involving recessive mutations, we used only those gnomAD variants that had been observed at least once in a homozygous state for the putatively benign variant group. Mutations were mapped to protein structures only considering those polypeptide chains with >90% sequence identity to a human protein over a region of at least 50 amino acid residues. Mutations were only mapped to structures where the residue of interest, as well as its adjacent neighbours, were the same as the human wild-type sequence. Molecular disease mechanism annotations were derived only considering genes that were annotated with an inheritance of 'AD' or 'AR' in OMIM.
Replication	Non-deterministic variant stability effect prediction scores were derived as averages of triplicate prediction runs. Other results are reproducible owing to the computational and deterministic nature of the dataset generation and analysis. All attempts at replication were successful.
Randomization	Group allocations were carried out based on pre-determined criteria outlined in the methods section.<br><br>Structural locations were classified as interior, surface or interface according to a classification previously established in Levy, E. D. (J. Mol. Biol., 2010). Interface residues show a solvent accessible surface area difference between the free subunit and full protein structure. Other residues with less than 25% relative solvent accessible surface area in the full structure were classified as interior, while the remainder was designated as surface.<br><br>Molecular disease mechanism annotations for genes were derived based upon information available in the OMIM and ClinGen databases. Only genes that were annotated with an inheritance of 'AD' in OMIM were considered for alternative molecular mechanism classification (haploinsufficient, dominant-negative or gain-of-function). Genes annotated as 'Sufficient evidence for dosage pathogenicity' in ClinGen were classified as 'haploinsufficient'. Next, all OMIM entries were searched for the keywords 'dominant negative', 'gain of function' and 'activating mutation'. Identified OMIM entries for genes were manually curated. If there was evidence in the OMIM entry that a pathogenic missense mutation was due to one of these mechanisms, then it was assigned as 'dominant-negative' or 'gain-of-function'. We attempted to minimize bias by only considering information available for all genes in the OMIM entries.
Blinding	Blinding was not possible due to the necessity for manual curation of the groups.

Reporting for specific materials, systems and methods

We require information from authors about some types of materials, experimental systems and methods used in many studies. Here, indicate whether each material, system or method listed is relevant to your study. If you are not sure if a list item applies to your research, read the appropriate section before selecting a response.

Materials & experimental systems

n/a	Involved in the study	
[x]	[ ]	Antibodies
[x]	[ ]	Eukaryotic cell lines
[x]	[ ]	Palaeontology and archaeology
[x]	[ ]	Animals and other organisms
[x]	[ ]	Human research participants
[x]	[ ]	Clinical data
[x]	[ ]	Dual use research of concern

Methods

n/a	Involved in the study	
[x]	[ ]	ChIP-seq
[x]	[ ]	Flow cytometry
[x]	[ ]	MRI-based neuroimaging
